# Genome-Wide Analysis of H3K27me3 in Porcine Embryonic Muscle Development

**DOI:** 10.3389/fcell.2021.739321

**Published:** 2021-11-05

**Authors:** Baohua Tan, Sheng Wang, Shanshan Wang, Jiekang Zeng, Linjun Hong, Zicong Li, Jie Yang, Gengyuan Cai, Enqin Zheng, Zhenfang Wu, Ting Gu

**Affiliations:** ^1^National Engineering Research Center for Breeding Swine Industry, College of Animal Science, South China Agricultural University, Guangzhou, China; ^2^Guangdong Provincial Key Laboratory of Agro-Animal Genomics and Molecular Breeding, College of Animal Science, South China Agricultural University, Guangzhou, China; ^3^Key Laboratory of Agricultural Animal Genetics, Breeding and Reproduction, Huazhong Agricultural University, Ministry of Education, Wuhan, China

**Keywords:** H3K27me3, skeletal muscle, embryonic development, pig, ChIP-seq

## Abstract

The trimethylation of histone H3 lysine 27 (H3K27me3) is one of the most important chromatin modifications, which is generally presented as a repressive mark in various biological processes. However, the dynamic and global-scale distribution of H3K27me3 during porcine embryonic muscle development remains unclear. Here, our study provided a comprehensive genome-wide view of H3K27me3 and analyzed the matching transcriptome in the skeletal muscles on days 33, 65, and 90 post-coitus from Duroc fetuses. Transcriptome analysis identified 4,124 differentially expressed genes (DEGs) and revealed the key transcriptional properties in three stages. We found that the global H3K27me3 levels continually increased during embryonic development, and the H3K27me3 level was negatively correlated with gene expression. The loss of H3K27me3 in the promoter was associated with the transcriptional activation of 856 DEGs in various processes, including skeletal muscle development, calcium signaling, and multiple metabolic pathways. We also identified for the first time that H3K27me3 could enrich in the promoter of genes, such as *DES*, *MYL1*, *TNNC1*, and *KLF5*, to negatively regulate gene expression in porcine satellite cells (PSCs). The loss of H3K27me3 could promote muscle cell differentiation. Taken together, this study provided the first genome-wide landscape of H3K27me3 in porcine embryonic muscle development. It revealed the complex and broad function of H3K27me3 in the regulation of embryonic muscle development from skeletal muscle morphogenesis to myofiber maturation.

## Introduction

Skeletal muscles represent the most abundant tissue of the body, and such muscles are essential for motion and support (Chal and Pourquié, 2017). Compromised muscle function can result in debilitating musculoskeletal disorders from developmental disorders to muscular dystrophies (Chal and Pourquié, 2017; Yue et al., 2021). Embryonic skeletal muscle development (embryonic myogenesis) predominantly determines the growth and mass of skeletal muscle. The total number of muscle fibers is basically fixed during embryonic myogenesis, which determines the postnatal fiber hypertrophy (Rehfeldt et al., 2008; Ali et al., 2021; Yue et al., 2021). In addition, impaired embryonic myogenesis has a long-lasting effect on postnatal muscle growth and animal performance, even after compensatory growth (Ali et al., 2021). Thus, research on embryonic muscle development is critical to reveal the genetic mechanisms affecting muscle development and improve meat production and animal health.

Embryonic myogenesis begins from the proliferation and differentiation of skeletal myogenic progenitors in somites. These cells lose the expression of the transcription factors (*Pax3* and *Pax7*) during the progressive specialization as the expression of myogenic regulatory factors (MRFs), including *Myf5*, *Myod*, *Myog*, and *Mrf4*, increases. Skeletal muscle formation continues with the differentiation of these specialized progenitors to form muscle fibers (Tu et al., 2016). Pig is an omnivorous, monogastric species with many anatomic and physiological similarities to human, which can serve as an animal model for muscle diseases (Pabst, 2020). Pig embryonic myogenesis is a complex process that consists of two growth waves: the first growth wave occurs during 30–55 days post coitus (dpc) when primary skeletal muscle fibers develop and provide the attached scaffold for the secondary skeletal muscle fibers; the second growth wave occurs from 50 dpc to 90 dpc, when the secondary muscle fibers form and increase in number by many times (Yue et al., 2021). The formation of myofibers ceases and the total number of myofibers is settled by 85-90 dpc (Ali et al., 2021). The continual advances of high-throughput sequencing methods in epigenetics allow lower input material and more accurate examination of the dynamic alterations to histone, DNA, and RNA methylation patterns (Barski et al., 2007; Dominissini et al., 2012; Wang et al., 2013; Abo Alrob and Lopaschuk, 2014). These techniques with increasingly analytical tools have contributed to the investigation of RNA splicing and transcriptional regulation in various processes, and the prediction and therapy of diseases (Heerboth et al., 2014; Gu et al., 2015; Bauer et al., 2016). Myogenesis in pig is regulated by various epigenetic regulations, such as noncoding RNA, RNA methylation, DNA methylation, and histone modifications (Begue et al., 2017; Kudou et al., 2017; Jin et al., 2018; Qin et al., 2019; Wang et al., 2019c; Yonamine et al., 2019). H3K27me3 is one of the most frequent histone modifications that govern chromatin structure and gene expression. EZH2 is the catalytic subunit of the polycomb repressive complex 2 (PRC2), which is responsible for H3K27me3 that leads to gene silencing. Increasing studies have indicated that H3K27me3 plays an important role in various processes of skeletal muscle development. H3K27me3 can regulate the differentiation of satellite cells by altering the gene expression of MRFs, such as *Myog* (Asp et al., 2011; Faralli et al., 2016; Adhikari and Davie, 2018). The deposition of H3K27me3 in the promoter of *Ccnd1* and *Ccne1* can inhibit the proliferation of muscle cells (Adhikari et al., 2019). H3K27me3 level increases during muscle regeneration, and the loss of H3K27me3 demethylase UTX or methylase EZH2 activity impairs regeneration and reduces the number of stem cells (Liu et al., 2013; Woodhouse et al., 2013; Faralli et al., 2016). The fiber area and muscle mass of conditional *EZH2* knockout mouse decrease as the global H3K27me3 level is significantly reduced (Woodhouse et al., 2013). During embryonic development, abnormal H3K27me3 causes embryonic lethality by knocking out demethylase *UTX* (Faralli et al., 2016). H3K27me3 can affect the acquisition and repression of defined cell lineage transcriptional programs of embryonic stem cells (Juan et al., 2016). A study on ovine skeletal muscle has presented genome-wide maps of H3K27me3 during the late stage of embryonic development and revealed the important role of H3K27me3 in the early development process and neuron function (Byrne et al., 2014). However, the regulation of H3K27me3 in the skeletal muscle of pigs during embryonic development remains poorly elucidated.

In this study, we performed RNA-seq and chromatin immunoprecipitation (ChIP)-seq to provide the first comprehensive analysis of H3K27me3 and gene expression profile during embryonic muscle development (d33, d65, and d90) in Duroc pig. Our analysis revealed the transcriptome characteristics in the three stages and investigated the dynamic changes of gene expression in muscle development. We highlighted the important role of H3K27me3 in regulating gene expression and identified the essential processes regulated by H3K27me3 during pig embryonic myogenesis. This study can provide the basis for studying and expanding the regulatory mechanisms of muscle fiber development.

## Materials and Methods

### Sample Collection

Duroc pigs (*Sus scrofa*) are widely used as terminal boars for its elite growth performance. In this study, Duroc fetuses were obtained from the pig breeding farm of Guangdong Wen’s Foodstuffs Group Co., Ltd. (Yunfu, China). Nine Duroc pig fetuses were divided into three groups, consisting of three fetuses on gestation day 33 (d33), three fetuses on gestation day 65 (d65), and three fetuses on gestation day 90 (d90). The three fetuses on the same gestation day were half-sibs for reducing the influence of individual differences. The longissimus muscle tissues were quickly sectioned in a 2-ml centrifuge tube and stored in liquid nitrogen.

### Cell Culture

The isolation of porcine satellite cells (PSCs) was performed as previously described (Wang et al., 2019a). For proliferation, PSCs were cultured in RPMI-1640 medium (11875119, Gibco, United States) containing 20% FBS (10099141, Gibco, United States), 1% non-essential amino acids (Gibco, United States), 0.5% chicken embryo extract (0928501, GEMINI, United States), 1% GlutaMax (35050061, Gibco, United States), 1% antibiotic–antimycotic (15140122, Gibco, United States), and 2.5 ng/ml bFGF (13256029, Gibco, United States) under moist air with 5% CO_2_ at 37°C. At about 70% confluence, DMEM high-glucose medium (12430054, Gibco, United States) supplemented with 2% horse serum (SH30074.03, HyClone, United States) and 1% antibiotic–antimycotic (15140122, Gibco, United States) was used to induce PSCs to differentiation. GSK343 (SML0766, SIGMA, United States) was dissolved to 2.5 μmol/L with DMSO (D4540, SIGMA, United States) and used for the depletion of H3K27me3 in PSCs. After 48-h incubation with 2.5 μmol/L GSK343 or vehicle 0.1% DMSO in proliferation, PSCs were induced to differentiation and continually treated for 36 h.

### Quantitative RT-PCR

Total RNA was extracted by using TRIzol reagent (15596018, Gibco, United States) according to the manufacturer’s instruction. PrimeScript RT reagent kit with gDNA Eraser (RR047A, Takara, Japan) was used to erase genomes and reverse transcription. The reaction mixture and condition for quantitative real-time PCR (RT-qPCR) was described in our published paper (Tan et al., 2020). The qPCR reaction was performed in Quant Studio 7 Flex Real-Time PCR System (Thermo Fisher Scientific, United States). All experiences were carried out with three fully independent biological replicates and three technical repeats. The relative gene expression level was calculated by the Ct (2^–ΔΔCt^) method. The primers are listed in Supplementary Table S5.

### Western Blotting

Porcine satellite cells or muscle tissues were lysed in RIPA buffer containing 1% (v/v) phenylmethylsulfonyl fluoride (PMSF) (ST505, Beyotime, China) to acquired total protein. Approximately 30 μg of protein of each sample was loaded on SDS-PAGE and transferred to PVDF membranes (IPVH85R, Millipore, United States). After blocking with 5% non-fat milk, the proteins in membranes were subjected to immunoblotting analysis with primary and secondary antibodies. Antibodies used in this study were as follows: anti-H3K27me3 (17-622, Millipore, United States), anti-beta-tubulin (GB11017, Servicebio, China), anti-MYOD (sc-760, Santa Cruz Biotechnology, United States), anti-MYH4 (A15293, ABclonal, United States), and HRP-conjugated Affinipure Goat Anti-Rabbit IgG (H+L) (SA00001-2, Proteintech, United States). The membranes were developed with ECL (WBULS0500, Millipore, United States) for visualization. ImageJ software was used to determine the bands’ signal intensities. The density value of each band was normalized by corresponding beta-tubulin density value.

### Immunofluorescence

Porcine satellite cells were cultured in a six-well plate and differentiated for 1.5 days. The immunofluorescence staining was performed according to our previous studies (Wang et al., 2019b). The antibodies and their dilutions were as follows: MYHC (sc-376157, Santa Cruz Biotechnology, United States) and secondary antibody (A0521, Beyotime, China). Cell nuclei were stained by using 4′,6-diamidino-2-phenylindole (DAPI; C1006, Beyotime, China). The cell differentiation index was calculated by the ratio of the number of nuclei in the myotubes to the total number of nuclei in one field of view.

### Chromatin Immunoprecipitation

Chromatin immunoprecipitation experiment was carried out by using ChIP kit (P2078, Beyotime, China) according to the recommended protocol. Briefly, 4 × 10^6^ PSCs were cultured to 90% confluence and fixed for 10 min at room template with 1% formaldehyde. The crosslinking was stopped by adding 125 mM glycine. Cell lysates were sheared by sonication in 1% SDS lysis buffer to generate chromatin fragments, followed in sequence with immunoprecipitation, reversal of cross-links, and DNA purification. Two micrograms of antibody H3K27me3 (17-622, Millipore, United States) or negative control IgG was used to each immunoprecipitated reaction. Fold enrichment was quantified using RT-qPCR. The primers are listed in Supplementary Table S5.

### RNA-Seq and Data Analysis

RNA-seq was performed using three biological replicates. Total RNA was isolated using the TRIzol reagent (15596018, Gibco, United States) according to the manufacturer’s protocol. Sequencing libraries were generated using NEBNext^®^ Ultra^TM^ RNA Library Prep Kit for Illumina^®^ (E7775, New England BioLabs, United States). Clean reads were obtained by removing reads containing adapter, reads containing ploy-N, and low-quality reads from raw data by using fastp (Chen et al., 2018). BWA mem (Li, 2013) was used to build the index and map the clean reads to reference genome of pig (Ensembl Sscrofa 11.1.94) with default parameters. The gene read counts were calculated by featureCounts (Liao et al., 2014) and normalized to the transcripts per kilobase million (TPM). Differential gene expression analysis between two groups was performed using the R package DESeq2 (Love et al., 2014). Gene ontology (GO) analyses, Kyoto Encyclopedia of Genes and Genomes (KEGG) pathway analyses, and Gene Set Enrichment Analyses (GSEA) were implemented by R package clusterProfiler (Yu et al., 2012). All gene sets for GSEA were downloaded from MSigDB (Subramanian et al., 2005). R package ggplot2 was contributed to the graphical representation (Ginestet, 2011). Gene with TPM ≥ 0.5 at least in one sample was defined as expressed genes. All genes were classed into four groups according to their expression in each stage: high level (TPM ≥ upper quartile), medium level (lower quartile < TPM < upper quartile), low level (TPM ≤ lower quartile), and silent (never expressed).

### Chromatin Immunoprecipitation-Sequencing and Data Analysis

Chromatin immunoprecipitation sample for sequencing was obtained by using the SimpleChIP Enzymatic Chromatin IP kit (9005, Cell Signaling Technologies, United States). Briefly, approximately 25 mg of skeletal muscle tissues was minced with a scalpel, harvested in 1 ml of PBS, cross-linked by 45 μl of 37% formaldehyde for 20 min at room temperature, and quenched with Glycine Solution for 5 min at room temperature. The suspended tissues were centrifuged 500 × *g* for 5 min at 4°C, washed two times in cold PBS, homogenized using a Dounce homogenizer, and incubated for 20 min at 37°C with micrococcal nuclease. Nuclei were destroyed by sonication, and the debris was removed by centrifugation. The clarified nuclear extracts were incubated overnight with 4 μg of H3K27me3 antibody (17-622, Millipore, United States) and immunoprecipitated with protein G magnetic beads. ChIP DNA library was constructed by Novogene Corporation (Beijing, China). Subsequently, 150-bp pair-end sequencing of sample was performed on Illumina platform. Library quality was determined with the Agilent Bioanalyzer 2100 system. Clean reads were obtained by removing reads containing adapter, reads containing ploy-N, and low-quality reads from raw data by using fastp (Chen et al., 2018). Clean reads were mapped to reference genome of pig (Ensembl Sscrofa 11.1) with default parameters by BWA mem (Li, 2013). Duplicated fragments, fragments with a mapping quality of less than 20, were removed by samtools (Danecek et al., 2021). The H3K27me3 peaks were identified by epic2 (Stovner and Sætrom, 2019) with a *q*-value threshold of 0.05 in broad model and >1-fold enrichment over the background. The R package ChIPseeker (Yu et al., 2015) was used to annotate genomic region of the peak and find the nearest genes around the peaks. The enrichment profiles of H3K27me3 and principal component analysis (PCA) of ChIP-seq data were performed using DeepTools (Ramírez et al., 2014). Read coverage for select genes was visualized in the Integrative Genomics Viewer (IGV) (Thorvaldsdóttir et al., 2013). The ChIP-seq peaks that were within −5 kb to 5 kb of transcription start sites (TSSs) were defined as promoter peaks. Read densities (RPKM) were defined as the normalized reads mapping in the promoter per kilobase million. The H3K27me3 level of gene is represented as normalized RPKM using IP/input ratio averaged in the two biological replicates.

### Statistical Analysis

Statistical analysis for the results of qPCR and Western blotting was performed by independent sample Student’s *t*-test in SPSS 20.0 software (IBM, Armonk, NY, United States). A *p*-value of less than 0.05 was considered as significant difference. Experiment results were presented as the mean ± standard error of mean (SEM).

## Results

### Gene Expression Dynamics During Porcine Embryonic Muscle Development

**FIGURE 1:**
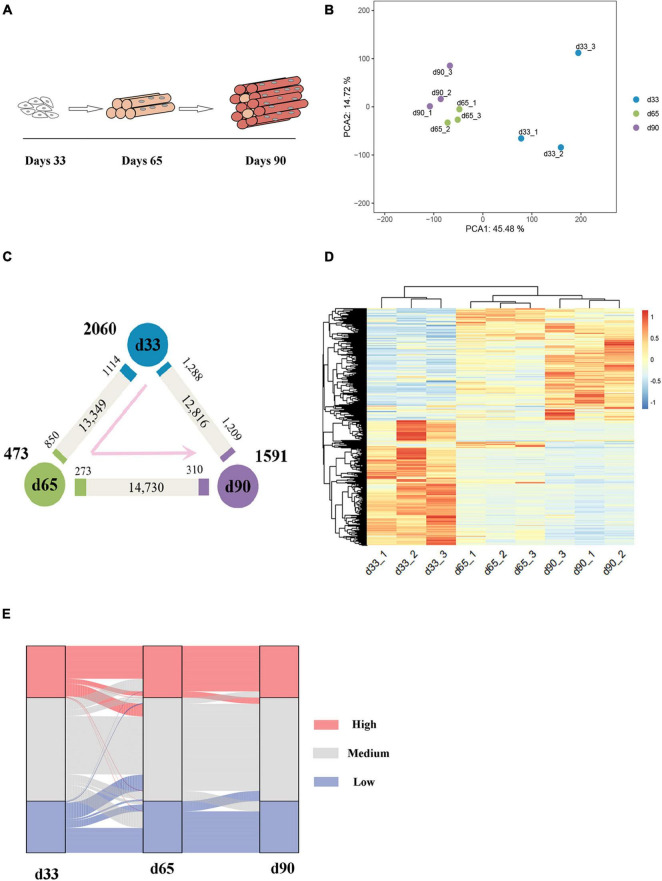
Gene expression dynamics during porcine embryonic muscle development. **(A)** Scheme for the three key stages in porcine embryonic muscle development. **(B)** The results of PCA on gene expression data. **(C)** Gene expression changes. The numbers of gene upregulated (*p*.adj ≤ 0.05, ≥2-fold change, marked by the corresponding colors) and unchanged (gray) at each stage compared with its neighboring stage are indicated. The numbers of genes expressed at the highest level in each stage are indicated in bold next to each stage. **(D)** The hierarchical clustering heatmap of DEGs. **(E)** The alluvial diagram of DEGs. High (TPM ≥ upper quartile); medium (lower quartile < TPM < upper quartile), low (TPM ≤ lower quartile) levels.

The transcriptomes of embryonic porcine longissimus muscle tissues were examined on d33, d65, and d90 to represent three critical time points of porcine embryonic development (Figure 1A and Supplementary Figure S1). Approximately 560 million reads with a unique mapped rate of above 90% were obtained (Supplementary Table S1). The close clustering of biological replicates in the PCA plots indicated high reproducible results (Figure 1B). Upon transcriptome analysis, we detected a total of 4,124 differentially expressed genes (DEGs) (Supplementary Table S2). We explored genes that were upregulated or downregulated among the successive stages and identified those expressed at the highest level in d33 (2,060, namely, “the d33 genes”), d65 (473, namely, “the d65 genes”), or d90 (1,591, namely, “the d90 genes”; Figure 1C). We further hierarchically clustered the expression across three stages and found that all DEGs were primarily clustered into two groups: (1) particularly high expression in d33 and (2) commonly high expression in d65 and d90 (Figure 1D). To preliminarily explore the biological processes with dynamic changes among the successive stages, we generated an alluvial diagram to reflect the degree of differences in expression and found DEGs with dramatical changes in expression (Figure 1E). The expression of DEGs slightly changed from d65 to d90, whereas 23 genes drastically changed between 33 days and 65 days (Supplementary Table S3). Some of these genes, such as *TMOD4* and *CAVIN4*, which are involved in the positive regulation of muscle development, were activated, whereas the expression of neural development-related genes, including *WNT6* and *CNTN2*, decreased. Collectively, these results showed the dynamic changes of gene expression during embryonic muscle development.

### Functional Enrichment Analysis of Differentially Expressed Genes

**FIGURE 2:**
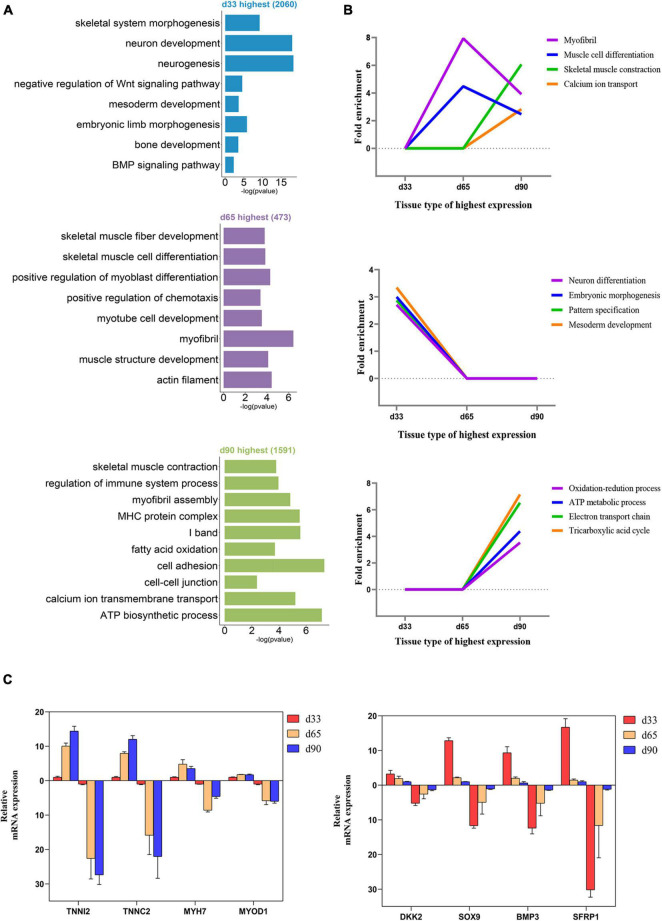
Functional enrichment analysis of differentially expressed genes. **(A)** The GO enrichment analysis of DEGs expressed at the highest level in the three stages. **(B)** Fold enrichment of genes for the presented GO terms. Fold enrichment is the proportion of term genes found in the DEGs list compared to the proportion of total term genes found in the background. **(C)** Validation of the expression of DEGs using RT-qPCR (the above of the *x*-axis represents the results of RT-qPCR; the below of the *x*-axis represents the results of RNA-seq).

We performed GO function category analysis to explore the function of DEGs (Supplementary Table S4). We revealed that the genes for neuron development, embryonic skeletal morphogenesis, and myogenic progenitor development were primarily enriched in the d33 genes, but they were depleted in the d65 genes (Figures 2A,B). By contrast, the genes, including *MYOD1*, *MYOG*, *MYH7*, and *TNNC1*, for skeletal muscle organ development, myofibril, and myoblast differentiation were prevalent among the d65 genes, which would contribute to the formation of skeletal muscle fiber (Figures 2A,B). The d65 and d90 genes were distinguished by their unique expression pattern in skeletal muscle development, metabolism, immune response, and cell–cell interaction properties. On the one hand, the d90 genes, such as *PLIN5* and *AK1*, were specifically enriched in the multiple metabolic processes (Figures 2A,B), including ATP biosynthesis, fatty acid oxidation, and tricarboxylic acid cycle, indicating that the promoting energy metabolism activity is required for organ maturation in the late stage of embryonic development. On the other hand, the d90 genes were characterized by those genes playing important roles in the skeletal muscle development. The genes for muscle cell differentiation and primary myofibril formation were more prevalent among the d65 genes, whereas the d90 genes were enriched in the development process from primary myofibrils to second myofibrils, including myofibril assembly, myotube development, and skeletal muscle contraction (Figures 2A,B). Several important genes were selected on the basis of our analysis results for validation. The relative expression levels of genes from RT-qPCR were consistent with those obtained from transcriptome sequencing (Figure 2C). These analyses provided a clear delineation of key transcriptional properties of the three stages during embryonic muscle development.

### Characteristic of H3K27me3 During Embryonic Development

**FIGURE 3:**
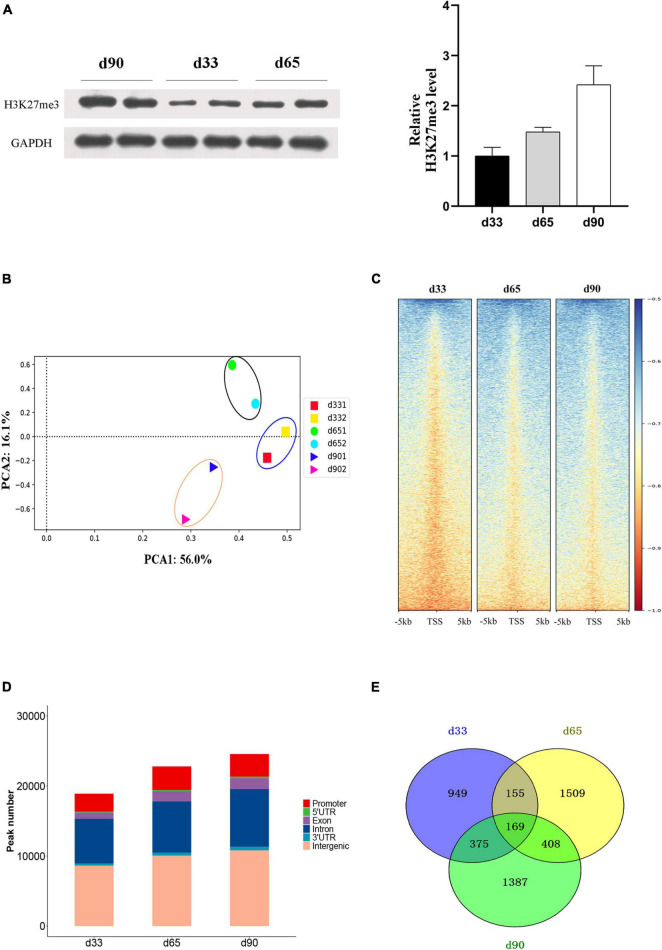
The characteristic of H3K27me3 during embryonic development. **(A)** Western blotting results showing the dynamics change of H3K27me3 in embryonic muscle development. The protein levels of H3K27me3 were quantified using ImageJ software. **(B)** Principal component analysis (PCA) of ChIP-seq data of H3K27me3 on d33, d65, and d90. The first and second principal components were depicted as *x*-axis and *y*-axis. **(C)** The enrichment heatmap of H3K27me3 around the 5 kb upstream or downstream of the TSS. **(D)** The genomic distribution of H3K27me3 region in three stages. **(E)** Numbers of shared and stage-specific genes associated with H3K27me3 peak in the promoter on d33, d65, and d90.

Studies have shown that H3K27me3 plays an important role in C2C12 myogenic differentiation (Asp et al., 2011), but few studies have focused on the function of H3K27me3 in porcine embryonic muscle development. Here, we first revealed that the gradual increase in the H3K27me3 level on d33, d65, and d90 indicated that H3K27me3 might regulate the development of porcine embryonic muscle (Figure 3A). The expression levels of genes encoding the core members of PRC2 (EZH2/EED/SUZ12), PRC1(RING1/CBX/BMI1), and demethylases (KDM6B/UTX) were highly dynamic, but they showed similar change pattern during prenatal myogenesis (Supplementary Figure S2). The highest expression in d33 and the sharp decrease from d33 to d65 of H3K27me3 demethylases might contribute to the low H3K27me3 level in d33 and increased H3K27me3 level during porcine embryonic development. We further performed ChIP-seq to gain epigenomic insights into the regulatory mechanisms of H3K27me3 underlying embryonic myogenesis. We obtained >25 million reads for each sample with the above 90% mapping rate, suggesting that the sequencing results of H3K27me3 were reliable (Supplementary Table S1). The PCA results revealed the clear separation of the replicates in different stages, and two biological replicates in each stage were clustered (Figure 3B). We calculated the average signals of H3K27me3 relative to the gene promoter region. Consistent with Western blotting, meta-plot showed that the H3K27me3 level in the region from TSS to TES increased during embryonic development (Supplementary Figure S3). Enrichment heatmap showed the close enrichment level around TSS during d65 to d90 (Figure 3C). Circos plot showed that H3K27me3 was particularly more enriched on the sex chromosome (Supplementary Figure S4).

We performed peak calling to identify the H3K27me3 peak and characterized the distribution of H3K27me3 in different stages. A total of 18,947, 22,815, and 26,888 peaks were found on d33, d65, and d90, respectively. The distribution of genomic regions modified by H3K27me3 was classified into six regions (promoter, exon, 3′UTR, 5′UTR, intron, and intergenic; Figure 3D). Consistent with the reported distribution model, we found that the H3K27me3 peak was primarily located in the intergenic and intronic regions (Han et al., 2020). The peaks in the promoter region (about 13.9% of all peaks) were used for further analysis. We found 949, 1,509, and 1,387 specific genes with promoter modified by H3K27me3 on d33, d65, and d90, respectively (Figure 3E). Finally, a total of 4,952 genes with a significant H3K27me3 peak in the promoter were identified, covering about 32% of 15,313 expressed genes, which suggested the broad regulation of H3K27me3 for embryonic muscle development. In summary, we observed that the global level of H3K27me3 dynamically changed across embryonic development and the H3K27me3 peak was primarily located in the intergenic and intronic regions.

### Association Analysis Between H3K27me3 and Gene Expression

**FIGURE 4:**
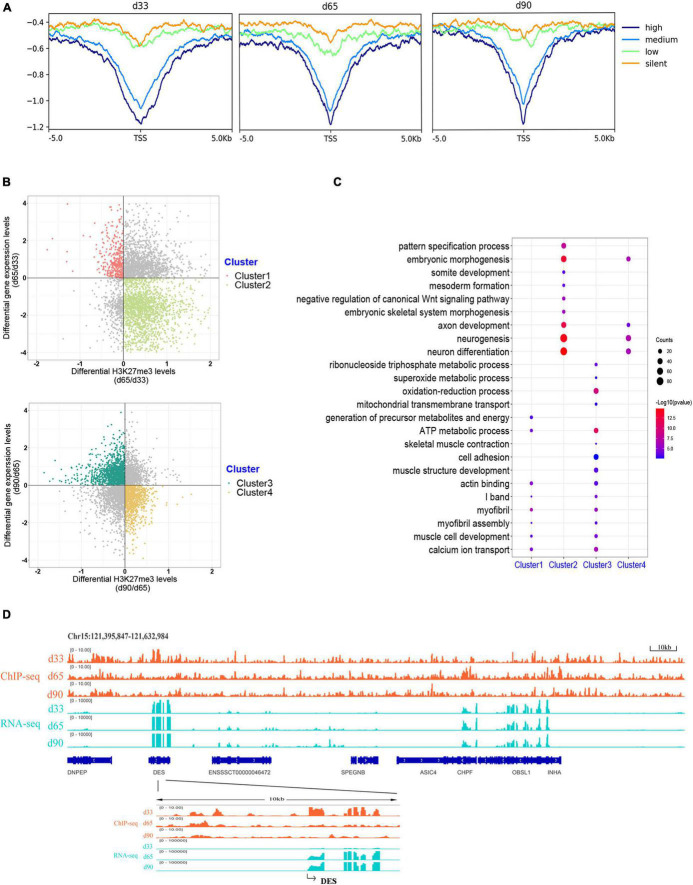
Association analysis between H3K27me3 and gene expression level. **(A)** The enrichment profile of H3K27me3 in all expressed gene. The *y*-axis was the average log2 fold change compared with control. High (TPM ≥ upper quartile; medium (lower quartile < TPM < upper quartile); low (TPM ≤ lower quartile); silent (never express). **(B)** Distribution of different expressed genes comparing the H3K27me3 levels and mRNA level. **(C)** The GO enrichment analysis of the DEGs exhibiting negative correlation between H3K27me3 and mRNA levels. **(D)** Genome browser tracks showing H3K27me3 ChIP-seq (orange) and RNA-seq (light blue) data at DES loci during embryonic muscle development. Promoter region (TSS ± 5 kb) of DES was zoomed in.

H3K27me3 is a well-known histone modification that generally represses gene transcription. We clustered all the expressed genes into four groups based on their expression to explore the correlation between H3K27me3 and gene level during embryonic development. The enrichment profile results showed that the gene expression was negatively correlated with H3K27me3 levels around the promoter in three stages (Figure 4A). These results indicated the critical role of the H3K27me3 in repressing gene expression during embryonic development.

We focused on the relationship between the gene expression and corresponding H3K27me3 level of 4,124 DEGs to explore whether gene expression was affected by H3K27me3. During d33 to d65, we found 391 upregulated genes with a decreasing H3K27me3 level and 1,907 downregulated genes with an increasing H3K27me3 level (clusters 1 and 2, respectively; Figure 4B). During d65 to d90, we found 1,243 upregulated genes with a decreasing H3K27me3 level and 916 downregulated genes with an increasing H3K27me3 level (clusters 3 and 4, respectively; Figure 4B). A total of 3,373 DEGs exhibited negative correlation between H3K27me3 and mRNA level at least in one period. Furthermore, we conducted GO analysis to reveal the molecular functions of 3,373 genes (Supplementary Table S6). We primarily focused on the dynamic change in H3K27me3 in the genes closely related to muscle development. Based on the GO analysis results of cluster 1 and 3 genes, the upregulated DEGs with loss deposition of H3K27me3 were enriched on the development of myofibril and muscle contraction (e.g., *MYH7*, *DES*, *MYL1*, *MYL3*, *TNNC1*, and *CAV3*). In addition, we found that numerous metabolism-related genes, such as *SLN*, *SLC4A1*, and *TPI1*, were activated with decreasing H3K27me3 level (Figure 4C). For cluster 2 and 4 genes, we found that the repressed state of biological processes, including myogenic progenitor development, embryonic morphogenesis, and neuron development, was associated with the gain deposition of H3K27me3 in the gene-related promoter (Figure 4C). Among these DEGs, *DES* is an early myogenic marker, and it is important to maintain maturation muscle cytoarchitecture (Soglia et al., 2019). Genome browser tracks of *DES* clearly revealed that the coverage of H3K27me3 around the promoter significantly decreased from d33 to d65 and remained at low levels in d90, whereas the contrary tendency of changes in the mRNA expression level was observed (Figure 4D). These results demonstrated that H3K27me3 in the promoter of DEGs was dynamically changed during embryonic muscle development, which likely regulated the initiation of myogenesis and the formation and maturation of myofibers by altering the expression of DEGs.

### Functional Enrichment Analysis of Genes With Promoter Modified by H3K27me3

**FIGURE 5:**
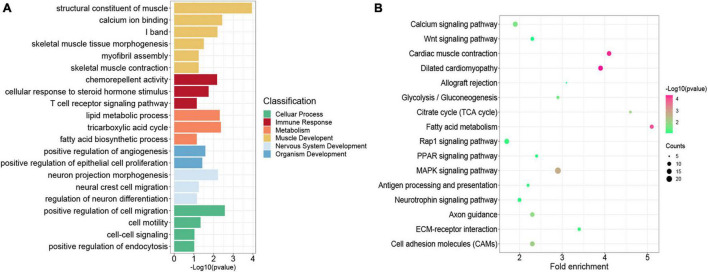
Functional enrichment analysis of genes modified by H3K27me3. **(A)** The GO enrichment analysis of the HDEGs. The colors represent the different classifications of GO terms. **(B)** The KEGG pathways analysis of HDEGs.

Previous studies indicated that H3K27me3 was not strong enough to control gene expression in some cases, although a significant H3K27me3 peak occurred in genes (Sawarkar and Paro, 2010; Grossniklaus and Paro, 2014). Thus, we overlapped all the 4,952 genes modified by H3K27me3 with the 3,373 DEGs exhibiting negative correlation between H3K27me3 and mRNA levels. Consequently, 856 genes (HDEGs) were modified with H3K27me3, and their H3K27me3 level in the promoter was negatively correlated with gene expression (Supplementary Table S7). They were defined as the key H3K27me3-modified gene and used for further analysis in this study. We performed GO and KEGG analysis of HDEGs to explore the biological process regulated by H3K27me3 (Supplementary Table S8). GO analysis indicated that HDEGs were primarily enriched during muscle development, immune response, epithelial and blood development, multiple metabolic processes including lipid biosynthetic process and fatty acid metabolic process, nerve system development, and cell–cell interaction (Figure 5A). KEGG pathway analysis identified several important pathways, including Wnt signaling pathway, MAPK signaling pathway, and calcium signaling pathway (Figure 5B). Wnt signaling pathway is vital in various embryonic muscle development processes, including somitogenesis, dermomyotome specification, and myogenesis (Chal and Pourquié, 2017; Girardi and Le Grand, 2018). We found that the genes in Wnt signaling pathway, such as *SFRP1*, *DKK2*, and *FZD5*, were regulated by H3K27me3, suggesting the important roles of H3K27me3 in regulating muscle development. Our study identified many HDEGs and revealed the essential regulatory processes of H3K27me3 during embryonic muscle development.

### Regulation of H3K27me3 During Embryonic Muscle Development

**FIGURE 6:**
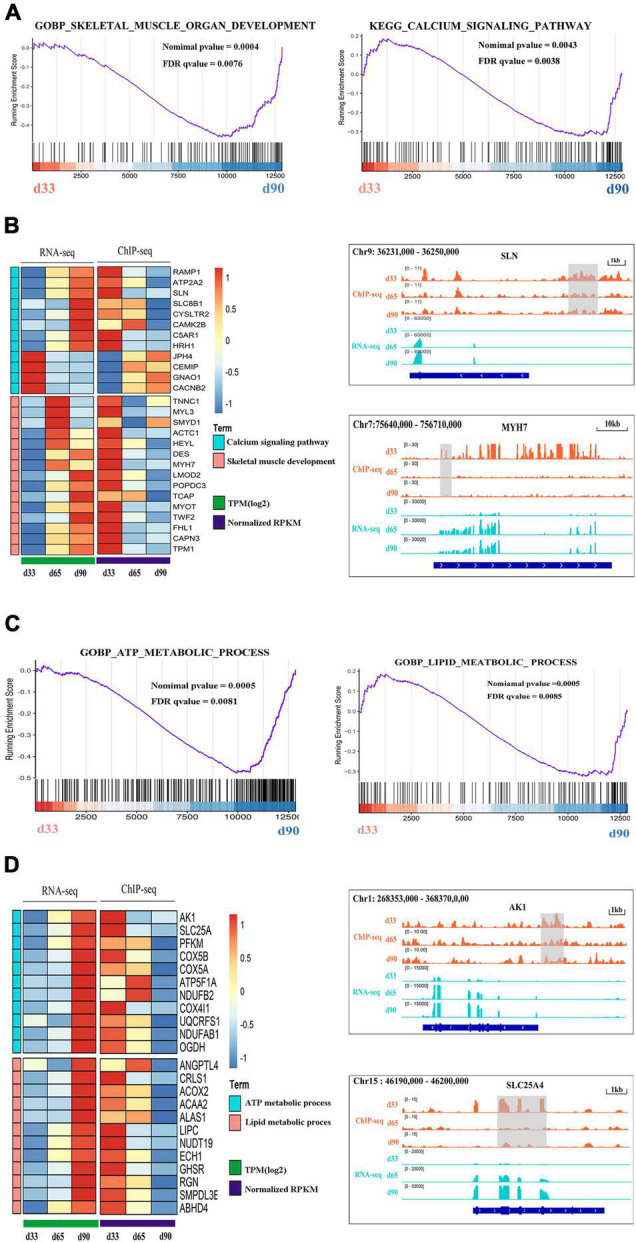
The regulation of H3K27me3 during embryonic muscle development. **(A)** GSEA plot of genes involved in the skeletal organ muscle development and calcium signaling pathway on d33 and d90. **(B)** The gene expression heatmap of HDEGs (left) in the skeletal organ muscle development and calcium signaling pathway. Genome browser tracks (right) showing the H3K27me3 ChIP-seq (orange) and RNA-seq (light blue) data at SLN and MYH7 loci during embryonic muscle development. H3K27me3 peak region was colored in gray. **(C)** GSEA plot of genes involved in the process of ATP metabolic and lipid metabolic on d33 and d90. **(D)** The gene expression heatmap of HDEGs (left) in the process of ATP metabolic and lipid metabolic. Genome browser tracks (right) showing the H3K27me3 ChIP-seq (orange) and RNA-seq (light blue) data at AK1 and SLC25A4 loci during embryonic muscle development. H3K27me3 peak region was colored in gray.

Previous studies indicated the important roles of metabolic homeostasis, and proper calcium transport activity in skeletal muscle development (Kraft et al., 2006; Tu et al., 2016). Here, we focused on the regulation of H3K27me3 for the HDEGs related to skeletal muscle organ development, metabolism, and calcium signaling pathways. Gene set enrichment analyses (GSEA) of porcine transcriptome revealed that the expression of the genes for skeletal muscle organ development was primarily higher in d33 than in d90 (Figure 6A). Some of these genes, including *MYH7*, *DES*, *LMOD2*, and *TCAP*, were modified by H3K27me3 (Figure 6B). These genes were upregulated with decreasing H3K27me3 level, which promoted the development of embryonic muscle after d33 (Figures 6A,B). In addition, we found that calcium signaling pathway was more activated during the late stage of embryonic development, although several genes still exhibited a high mRNA expression level at early stages (Figures 6A,B). With the promotion of the expression of myogenic genes, the transcription inhibition of genes, including *RAMP1*, *HRH1*, and *ATP2P2*, in the calcium signaling pathway disappeared as H3K27me3 in the promoter decreased after d33 (Figure 6B). On the contrary, some genes, such as *JPH4* and *CEMP*, were repressed after d33 (Figure 6B). These results suggested the complex regulatory mechanism of H3K27me3 presented in the regulation of calcium transport in muscle development. In addition, metabolism programming as a novel regulator of skeletal muscle development attracts lots of attention (Ryall, 2013). Thus, we analyzed the regulation of H3K27me3 in the ATP and lipid metabolic process. The GSEA results showed that the expression of the genes related to metabolism was higher in d90 than in d33, suggesting the high energy requirement in the later embryonic stages (Figure 6C). The read coverage of two typical genes, *AK1* and *SLC25A4*, was shown in IGV (Figure 6D). Our study indicated that the regulation of H3K27me3 for embryonic muscle development cooperated with the control for the metabolism and calcium signaling pathway.

### Exploration of H3K27me3 Function in Muscle Development *in vitro*

**FIGURE 7:**
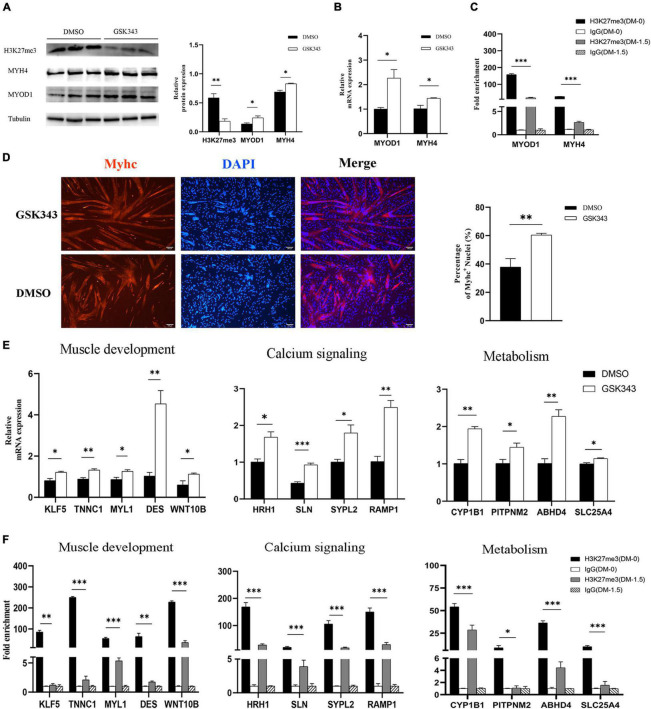
The exploration of H3K27me3 function in muscle development *in vitro*. **(A)** Western blotting results showing that the relative protein expression of MYOD1 and MYH4 was increased after GSK343 treatment on day 1.5 post differentiation compared with controls (0.1% DMSO). The protein levels of these genes were quantified using ImageJ software. **(B)** RT-qPCR results showing that the relative mRNA expression of MYOD1 and MYH4 was increased after GSK343 treatment on day 1.5 post differentiation compared with controls (0.1% DMSO). **(C)** ChIP-qPCR results showing that the enrichment of H3K27me3 in the MYOD1 and MYH4 were decreased following proliferation to differentiation. **(D)** Immunofluorescence staining of MYHC showing significant upregulation of PSCs differentiation after GSK343 treatment on day 1.5 post differentiation compared with controls (0.1% DMSO). The number of MYHC^+^ cells was quantified using ImageJ software. **(E)** RT-qPCR results showing that the mRNA expression of selected HDEGs was increased after GSK343 treatment on day 1.5 post differentiation compared with controls (0.1% DMSO). **(F)** ChIP-qPCR results showing that the enrichment of H3K27me3 at the promoter of selected HDEGs were decreased following proliferation to differentiation. All values represent the mean ± SEM of three independent experiments. ^∗^*p* < 0.05, ^∗∗^*p* < 0.01, ^∗∗∗^*p* < 0.001, ns indicates not significant.

Numerous studies have demonstrated that embryonic myogenesis and regeneration in adult skeletal muscle share many transcription factors and signaling molecules (Bentzinger et al., 2012). Satellite cells are a heterogeneous population of stem and progenitor muscle cells, which are capable of self-renewal and differentiation during muscle regeneration (Wang and Rudnicki, 2011). Thus, we used porcine satellite cells (PSCs) to verify the function of H3K27me3 *in vitro*. Instead of blocking the expression of EZH2, GSK343 molecule can directly inhibit enzymatic activity of EZH2 to reduce H3K27me3 through the *S*-adenosyl-L-methionine-competitive pathway (Liu et al., 2015). We used GSK343 to diminish the modification level of H3K27me3 in PSCs during cell differentiation. Western blotting result showed a significant decrease of H3K27me3 after GSK343 treatment in PSCs (Figure 7A). Myogenic marker genes (*MYOD1* and *MYH4*) were upregulated in 1.5d differentiated cells at mRNA and protein levels after the depletion of H3K27me3 (Figures 7A,B). We performed ChIP assay and found that *MYOD1* and *MYH4* were targeted by H3K27me3 following proliferation to differentiation (Figure 7C). Immunofluorescence staining of terminal differentiation marker (MYHC) revealed an increased number of MYHC^+^ cells, suggesting that H3K27me3 depletion could promote myogenic differentiation (Figure 7D). Collectively, these results indicated that H3K27me3 could target myogenic genes to reduce gene expression and regulate myogenic differentiation. Then, we selected several HDEGs involved in skeletal muscle development, calcium signaling pathways, and metabolism to detect the effect of H3K27me3 on these genes. *WNT10B* is modified by H3K27me3 and used as positive control in this study (Zhao et al., 2018). RT-qPCR results showed that genes such as *TNNC1*, *MYL1*, and *DES* for the positive regulation of muscle development; genes such as *ABHD4* and *CYP1B1* for metabolic process; and genes such as *SLN* and *RAMP1* for calcium transport were upregulated after GSK343 treatment (Figure 7E). ChIP-qPCR revealed that H3K27me3 was significantly enriched in the promoter of these genes, and the enrichment of H3K27me3 decreased during differentiation (Figure 7F). Furthermore, the H3K27me3 level had a strong negative correlation with mRNA expression levels following proliferation to differentiation (Supplementary Figure S5). In summary, these results demonstrated the promoting differentiation of PSCs after the depletion of H3K27me3 and verified the function of H3K27me3 in the regulation of muscle development, multiple metabolism, and calcium signaling pathway.

## Discussion

Skeletal muscle is a highly complex and heterogeneous tissue with a multitude of functions in the organism (Bentzinger et al., 2012). Embryonic muscle development shares a common process consisting of the proliferation of mesodermal stem cells, and progressive specialization into skeletal muscle progenitors, followed by the differentiation of muscle cells and the spatial arrangement of muscle cells to form the primary musculature. Furthermore, muscle cells initially fuse into multinucleated nascent myotubes and then further fuse into myofibers (Tu et al., 2016). A broad spectrum of signaling molecules instructs myogenesis during embryonic development. In this study, we focused on the regulatory mechanism of repressive histone modification, H3K27me3, in the development of porcine embryonic muscle. We comprehensively analyzed the transcriptome and chromatin modification of muscle tissues at specific stages and revealed the essential biological processes regulated by H3K27me3.

Our study initially investigated the dynamic change in gene expression at d33, d65 and d90 of porcine embryonic muscle development. We found that myogenic progenitor development and embryonic skeletal system morphogenesis were still activated on d33. During embryonic development, Wnt signals control the expression of MRFs and participate in myogenic lineage progression (von Maltzahn et al., 2012; Pan et al., 2015). On d33, the negative regulation of Wnt signaling pathway genes, including *WNT5A* and *DACT3*, were highly expressed, which can inhibit myogenesis by decreasing the gene expression of *MYOD1* (Adhikari and Davie, 2018). The MRFs (e.g., *MYOD1* and *MYOG*) and myofibril-formation-related genes (e.g., *TNNC1* and *MYH7*) were highly expressed on d65. This finding suggested the dynamic activation of the terminal differentiation of muscle cells, myotube fusion, and myofiber formation from d33 to d65. On d90, we found that the highly expressed genes were more enriched in myofibril assembly, muscle contraction, and associated calcium signaling pathway rather than the increasing myofibril formation and muscle differentiation. These results suggested that the period of the formation of primary muscle fibers was from d33 to d65, whereas the period of the development of second muscle fibers and muscle function was from d65 to d90 (e.g., muscle contraction). This finding was similar to that of the previous reports in pig (Picard et al., 2010; Qin et al., 2013). We observed that the changes in gene transcription from d33 to d65 were more dramatic compared with those from d65 to d90, which indicated that the regulatory mechanisms of the primary myofibers might be more complicated than those of second myofibers. We revealed the characteristics of the transcriptome on d33, d65, and d90 and demonstrated that these stages were suitable for the further investigation on the regulation of H3K27me3 for embryonic porcine muscle development.

The epigenetic mark H3K27me3 is one of the repressive histone modifications. Genome-wide mapping has revealed that H3K27me3 occupies in a large set of genes related to cell fate and embryonic development, including developmental transcription factors (such as Hox genes) and cell-surface or extracellular proteins (such as Wnt) (Boyer et al., 2006; Gao et al., 2010). Previous studies indicated the important roles of H3K27me3 in the regulation of muscle cells from proliferation to differentiation (Asp et al., 2011; Adhikari and Davie, 2018; Wang et al., 2019a). In our study, we found that the H3K27me3 level progressively increased during porcine embryonic development from 33d to 90d. The H3K27me3 levels around the TSS of all the expressed genes were negatively correlated with the gene expression levels at all stages. This result suggested that H3K27me3 might widely participate in the regulation of gene expression during embryonic muscle development. We obtained 856 DEGs, which possessed H3K27me3 enrichment peak, and the changes in the modification level were negatively correlated with gene expression. The functional enrichment analysis indicated the diverse function of these genes, implying that the effects of H3K27me3 covered a broad spectrum to modulate different physiological and developmental mechanisms during embryonic myogenesis. Previous studies pointed out that H3K27me3 always colocalized with activating histone modification H3K4me3 during embryonic development (Pan et al., 2007; Liu et al., 2016, 2019). This special modified state, consisting of two interrelated histone marks, is known as the “bivalent domain,” which could rapidly activate or inhibit gene transcription through the “winner-take-all” principle (Cui et al., 2012). In this study, 212 DEGs that were enriched by H3K27me3 but were not correlated with loss of transcript might be caused by the existence of bivalent domain in the gene body. This finding revealed the complex regulatory mechanism of H3K27me3 in governing gene expression during porcine embryonic development. We reported that muscle-fiber-formation-related genes, such as *MYH7*, *MYL3*, *TNNC1*, and *KLF5*, were modified by H3K27me3. These genes were activated to promote myofibril formation as the H3K27me3 level in the promoter decreased after d33. Kruppel-like factor 5 (*KLF5*), a zinc-finger transcription factor, is involved in the regulation of muscle differentiation coordinated with myogenic transcription factors such as *Myod* and *Mef2* (Hayashi et al., 2016). Previous studies demonstrated that the Wnt antagonist *SFRP1* can block *MYOD1* by inhibiting the translocation of β-catenin to the nucleus (Adhikari and Davie, 2018). Our studies indicated that *SFRP1* was modified by H3K27me3 and inhibited to express at d65. These results indicated that H3K27me3 could regulate muscle cell differentiation by indirectly regulating myogenic regulatory factors. In PSCs, we revealed that myogenic marker genes, *MYH4* and *MYOD1*, were targeted by H3K27me3, which was consistent with previous studies (Adhikari and Davie, 2018; Wang et al., 2020). The depletion of H3K27me3 could promote muscle cell differentiation by increasing the expression level of myogenic marker genes and the number of MYHC^+^ cells. These findings were similar with the previous studies (Caretti et al., 2004; Seenundun et al., 2010; Asp et al., 2011). Therefore, these data suggested that porcine myogenesis was required for the regulation of H3K27me3 to properly control gene expression.

Many of the myogenic factors that are operated during embryonic development are recruited for the growth of mature or maturing skeletal muscles, but myogenesis and plasticity are distinguished by different intrinsic and extrinsic environments and distinct competencies of cells. As a ubiquitous intracellular signal factor that regulates a series of cellular processes, Ca^2+^ participates in the regulation of muscle formation, growth, and regeneration (Tu et al., 2016). The identified molecular mechanisms underlying Ca^2+^ participation in muscle development include the regulation of stored–operated calcium entry (Stiber et al., 2008; Seth et al., 2012) and a number of signaling elements immediately downstream of the Ca^2+^ signal (Lu et al., 2000a, b). Our analysis showed the important role of H3K27me3 in the regulation of calcium signaling pathways, which are consistent with observation in the alcohol use disorder and chicken skin (Chang et al., 2015; Gavin et al., 2018). We first identified several genes, such as *SLN*, *RAMP1*, and *HRH1*, for calcium signaling pathways were regulated by H3K27me3 during embryonic muscle development. Sarcolipin (SLN), an inhibitor of the sarco/endoplasmic reticulum (SR) calcium pump, can reduce the Ca^2+^ content of SR and delay muscle differentiation (Seth et al., 2012). Our study demonstrated that H3K27me3 deposition is responsible for the repressed gene expression of *SLN* from d33 to d65, which promotes muscle cell differentiation. Mitogen-activated protein kinases (MAPK) signaling pathway is one of the downstream signaling of Ca^2+^ signal, which functions in the muscle progenitor proliferation and myoblast differentiation by, respectively, regulating the gene expression of *Ccnd1* and MRFs (Bennett and Tonks, 1997; Zetser et al., 2001). Based on the pathway analysis of H3K27me3, we found that MAPK signaling emerged as an overrepresented pathway, suggesting that the coordinating regulation with Ca^2+^ signaling in the development of skeletal muscle is also affected by H3K27me3 modification. These findings indicated the complex and broad functions of H3K27me3 in the regulation of spatiotemporal changes in Ca^2+^ dynamics and their signaling partners in embryonic muscle development.

Similar to all other cell types, muscle cells require energy to carry out the reactions necessary for life. The predominant source of cellular energy used to drive enzymatic reactions is the breakdown of ATP into ADP or AMP and inorganic phosphate (Ryall, 2013). ATP is primarily produced in the mitochondria *via* oxidative phosphorylation (OXPHOS). The two-carbon units are continuously eliminated from the β-position of fatty acyl-CoA molecule, thereby producing acetyl CoA to sustain OXPHOS and tricarboxylic acid cycle in cells during lipid oxidation (Yan et al., 2019). Our analysis found that multiple metabolic processes were more dramatic at the late stage of embryonic development, which were characterized by the high expression of genes for ATP metabolism and lipid metabolism. These results were associated with the fact that the density of mitochondria, and oxidative phosphorylation activity in muscle tissue dramatically increased following differentiation and fusion into myotube (Kraft et al., 2006). Few studies have focused on the relationships between H3K27me3 and metabolism during embryonic myogenesis. Our study found that H3K27me3 played complex and broad roles in the regulation of H3K27me3 in multiple metabolic processes and pathways. We identified that numerous metabolism-related genes, including *ABHD4* and *PORCN* for lipid metabolic process and *AK1* and *SLC25A4* for ATP metabolic process, were regulated by H3K27me3. *SLC25A4* is a mitochondrial ADP/ATP carrier, which is responsible for the ATP export from the mitochondria to the cytoplasm and provides energy to cells (Clémençon et al., 2013). We observed that the gene expression of *SLC25A4* was inhibited by H3K27me3 on d33 and upregulated in contracted muscle fiber to satisfy the high-energy-consuming reactions. In addition, our study showed that H3K27me3 was marked in the genes for glycolysis that is necessary for the differentiation of C2C12 cells (Fulco et al., 2008). Therefore, H3K27me3 could control the intensity of metabolism activity by regulating the proper transcript programming of metabolism-related genes during embryonic muscle development.

## Conclusion

In our study, we provided the first genome-wide profiling of H3K27me3 during porcine embryonic muscle development and revealed its important role in the calcium signaling pathway and metabolism coordinated with the regulation of myogenesis. This study could serve as the basis for analyzing not only animal breeding, human muscle disease, and embryonic development but also the regulatory mechanism of H3K27me3.

## Data Availability Statement

The datasets presented in this study can be found in online repositories. The names of the repository/repositories and accession number(s) can be found below: https://www.ncbi.nlm.nih.gov/sra/, PRJNA556496 and https://www.ncbi.nlm.nih.gov/sra/, PRJNA741351.

## Ethics Statement

The animal study was reviewed and approved by Animal Care Committee of South China Agricultural University, Guangzhou, China.

## Author Contributions

TG, SW, and BT conceived and designed the research and wrote and revised the manuscript. BT and SW conducted the sequencing data analysis. BT, JZ, and SSW performed the experiments. TG, LH, ZL, JY, and GC collected the samples. EZ administered the project. TG and ZW applied for the funding. All authors have read and approved the final submitted manuscript.

## Conflict of Interest

The authors declare that the research was conducted in the absence of any commercial or financial relationships that could be construed as a potential conflict of interest.

## Publisher’s Note

All claims expressed in this article are solely those of the authors and do not necessarily represent those of their affiliated organizations, or those of the publisher, the editors and the reviewers. Any product that may be evaluated in this article, or claim that may be made by its manufacturer, is not guaranteed or endorsed by the publisher.
