# Supplementary material for: Genome-Wide Analysis of H3K27me3 in Porcine Embryonic Muscle Development
Source: Front Cell Dev Biol. 2021 Nov 5;9:739321. doi: 10.3389/fcell.2021.739321 (PMC8602352; doi:10.3389/fcell.2021.739321)
Supplement: Supplementary file 9 [file Data_Sheet_9.docx]

Supplementary Figure

## Supplementary Figure


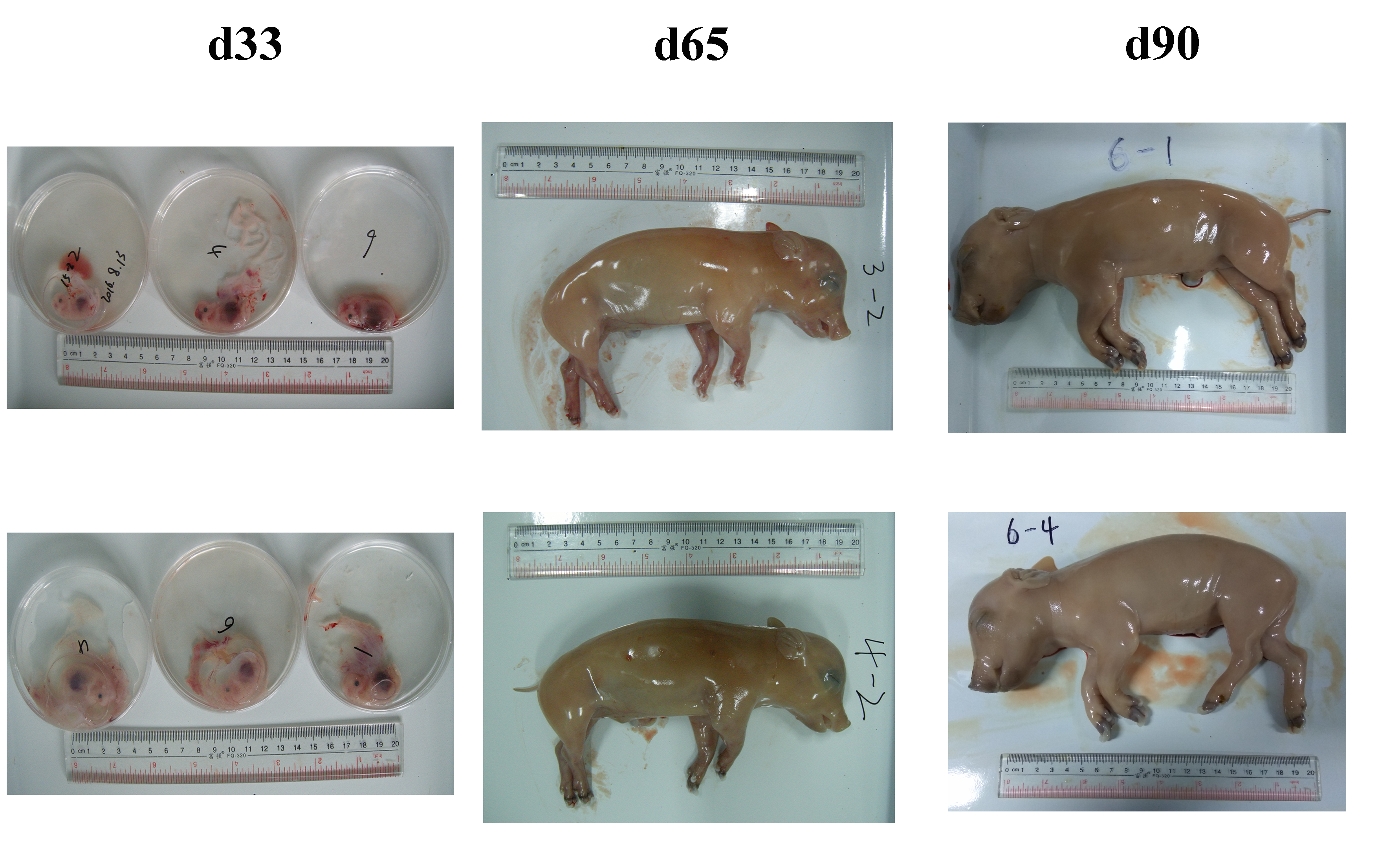


**Supplementary Figure 1.** The photos of fetal pig on d33, d65, and d90.

## Supplementary Figure


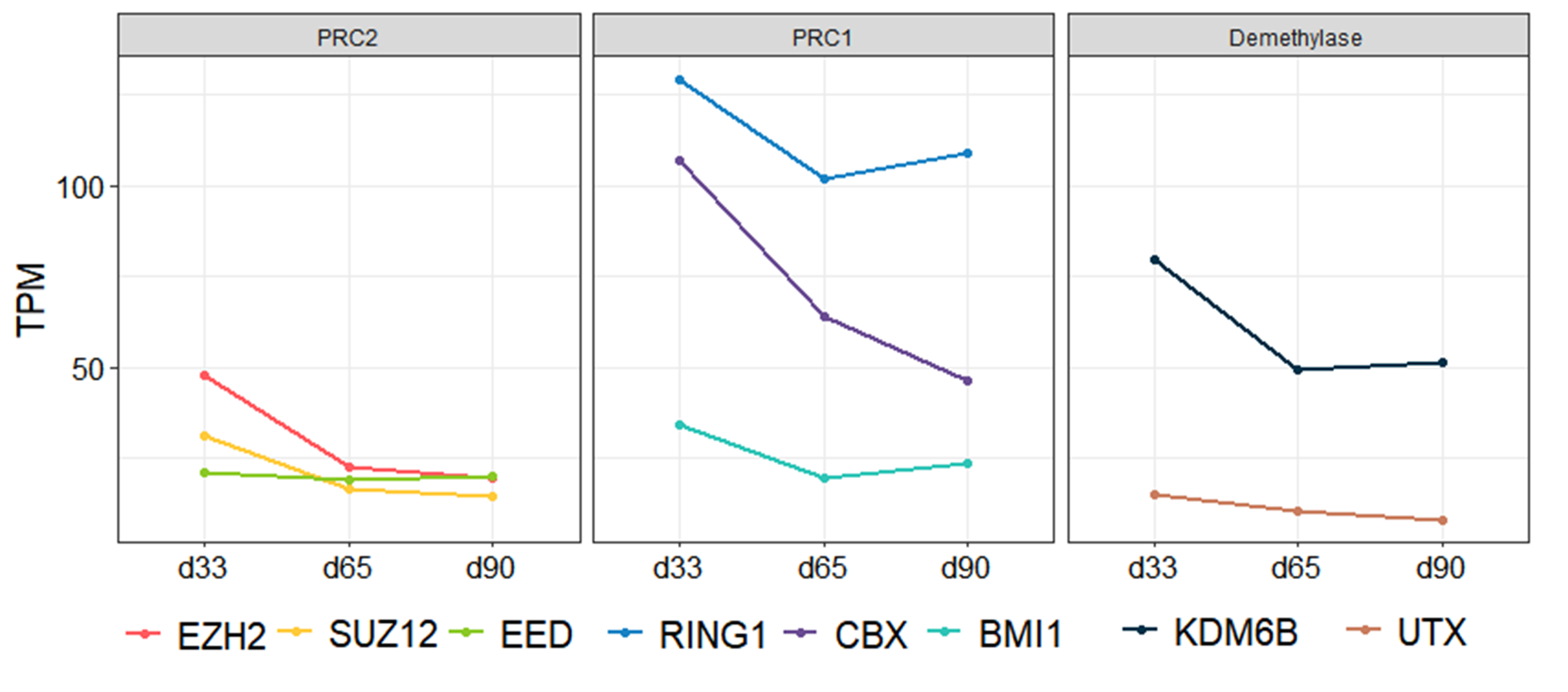


**Supplementary Figure 2.** Expression of the genes coding for the PRC2 (EZH2/EED/SUZ12), PRC1(RING1/CBX/BMI1) and H3K27me3 demethylases (KDM6B /UTX) .

## Supplementary Figure


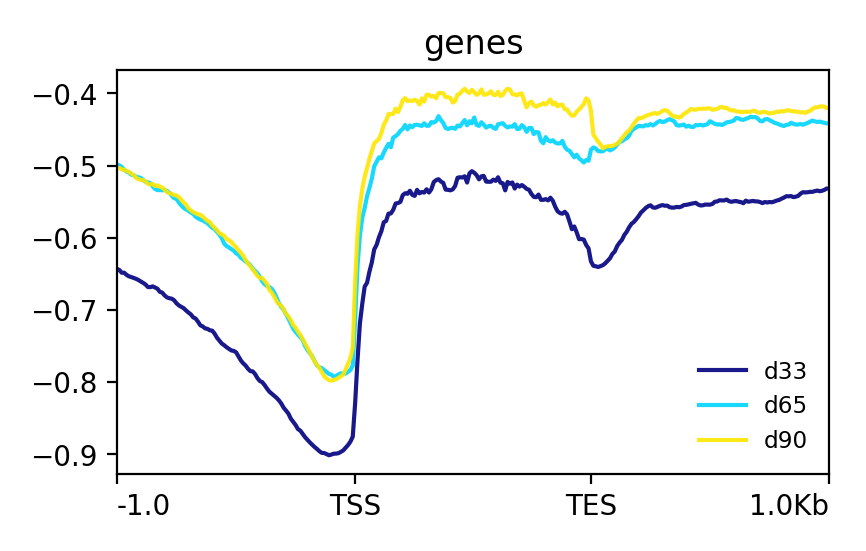


**Supplementary Figure 3.** Meta plot showing H3K27me3 level at genic region with 1kb upstream and 1kb downstream in all genes. The y-axis was the average log2 fold change compared with control.

## Supplementary Figure


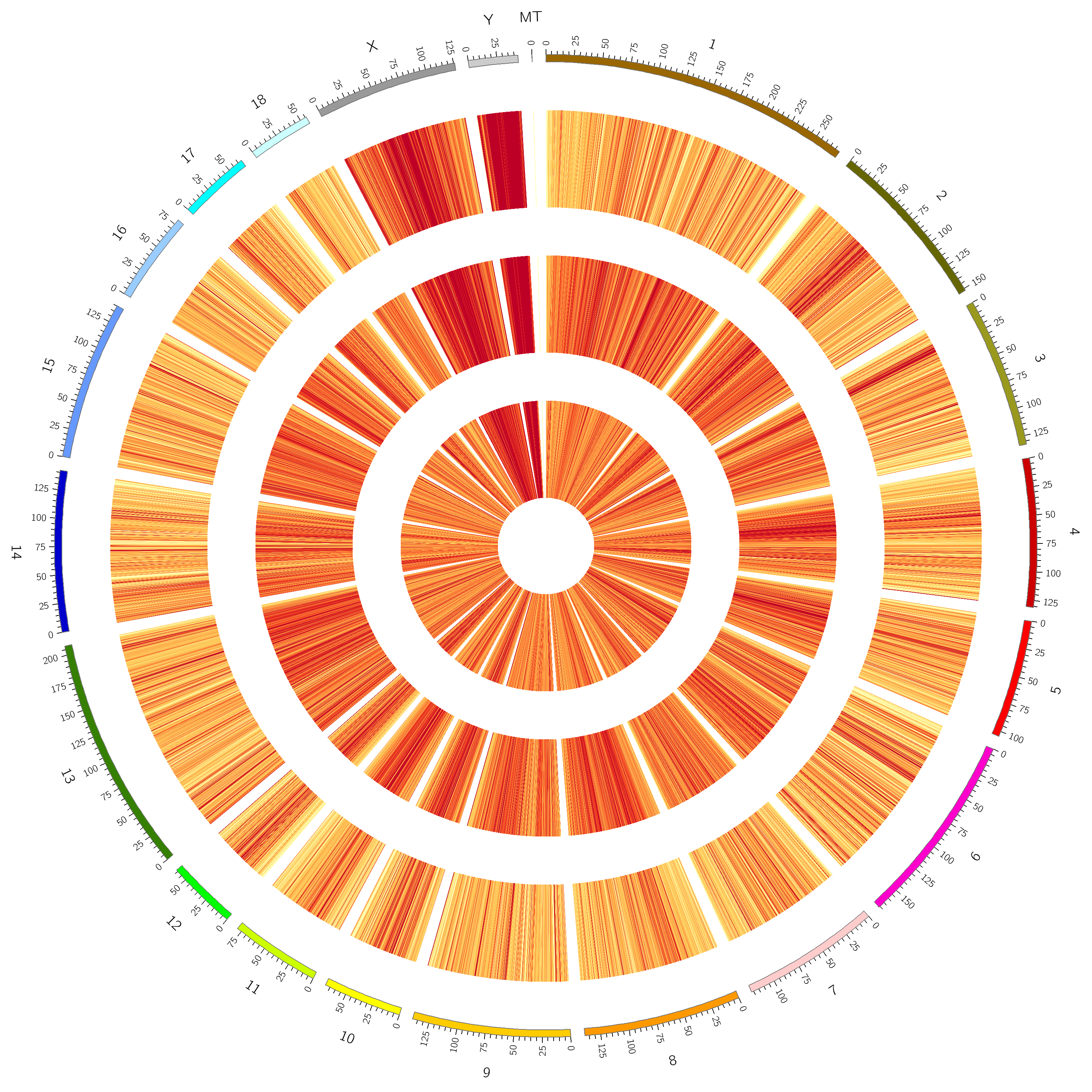


**Supplementary Figure 4.**  Circos-plot of H3K27me3 level corresponding to the chromosomal locations on d33 (outermost ring), d65 (middle ring), and d90 (innermost ring).

## Supplementary Figure


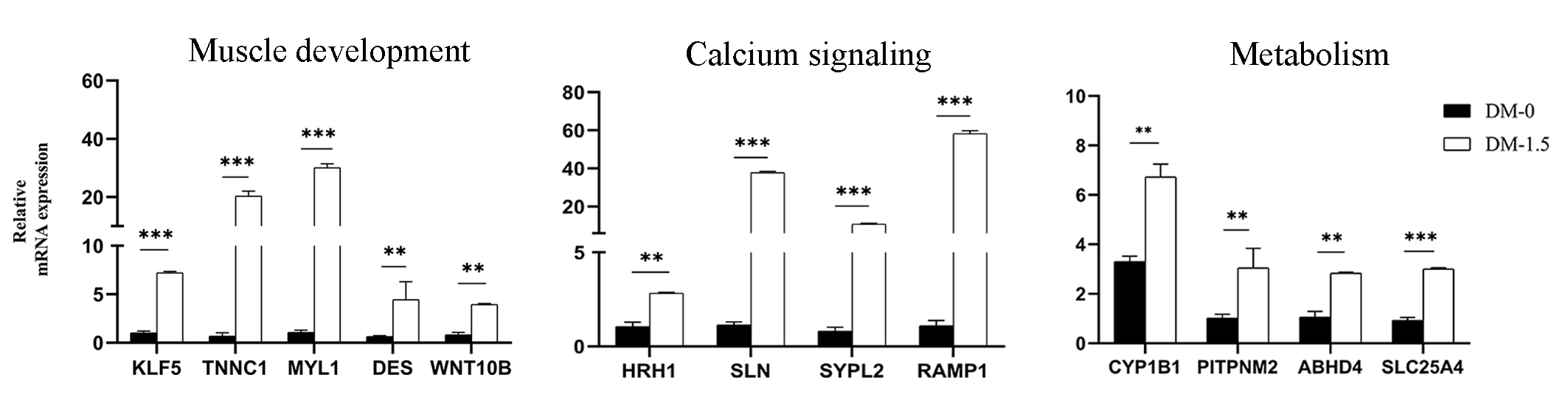


**Supplementary Figure 5.** RT-qPCR results showing that the relative mRNA expression of genes was significantly increased from proliferation to day 1.5 post differentiation of PSCs.
